# Severe pre-treatment attrition in the care cascade: clinical outcomes and risk factors among older MDR/RR-TB patients in China

**DOI:** 10.3389/fpubh.2026.1883068

**Published:** 2026-09-16

**Authors:** Fangjing Zhou, Shanshan Huang, Huiying Feng, Jianwei Li, Huizhong Wu, Yuhui Chen, Wenpei Wen, Lebin Liu

**Affiliations:** Public Health Medical Center of Guangdong Province, Guangzhou, China

**Keywords:** aged, multidrug-resistant tuberculosis, retrospective studies, rifampicin-resistant tuberculosis, treatment cascade

## Abstract

**Background:**

The rising global burden of multidrug- and rifampicin-resistant tuberculosis (MDR/RR-TB) coincides with rapid population aging. Older patients face unique challenges, yet age-stratified analyses of the MDR/RR-TB care cascade are lacking, obscuring the specific barriers they encounter.

**Objective:**

This study aimed to characterize the treatment cascade and identify factors associated with attrition among older MDR/RR-TB patients in China.

**Methods:**

We conducted a retrospective cohort study of 667 MDR/RR-TB patients aged ≥65 years in Guangdong Province, southern China (2010–2022). Using data from the national Tuberculosis Information Management System (TIMS), we constructed a care cascade to quantify attrition across four stages (diagnosis, treatment initiation, retention at 6 months, and treatment success) and three inter-stage gaps (attrition between successive stages). Separate multivariable logistic regression models identified factors associated with failure to initiate treatment and with poor outcomes.

**Results:**

Of the 667 diagnosed patients (median age 70), 500 (74.96%) initiated treatment, 406 (60.87% of diagnosed cases, 81.2% of treated cases) were retained at 6 months, and only 268 (40.18% of diagnosed cases, 53.6% of treated cases) achieved treatment success. Multivariable analysis showed that workers/farmers (*a*OR = 1.87, 95% CI: 1.24–2.84), diagnosis by conventional DST (*a*OR = 9.34, 95% CI: 4.52–21.64), diagnosis before 2016 (*a*OR = 1.91, 95% CI: 1.18–3.08), and older age (*a*OR = 1.05 per year, 95% CI: 1.01–1.08) were independently associated with failure to initiate treatment; prior TB treatment history was protective (*a*OR = 0.42, 95% CI: 0.25–0.72). Among treatment initiators, older age (*a*OR = 1.06 per year, 95% CI: 1.02–1.09) and diagnosis before 2016 (*a*OR = 1.88, 95% CI: 1.06–3.39) were independent risk factors for poor outcomes.

**Conclusion:**

The MDR/RR-TB care cascade for older patients in China is marked by severe pre-treatment attrition. Targeted interventions, including scaling up rapid molecular testing, providing targeted support (e.g., financial subsidies, home-based care, and flexible treatment schedules) for high-risk workers, and implementing geriatric-adapted follow-up, and expanding active case finding, are urgently needed to protect this vulnerable population and to close this equity gap.

## 1 Introduction

Tuberculosis (TB) remains a leading global cause of death from a single infectious agent, with an estimated 10.7 million new cases and 1.23 million deaths reported worldwide in 2024 (1). The burden of TB is critically compounded by the relentless emergence and spread of drug-resistant forms, primarily multidrug- and rifampicin-resistant tuberculosis (MDR/RR-TB). The 2025 World Health Organization (WHO) Global Tuberculosis Report estimated 390,000 incident cases of MDR/RR-TB, yet only 71% of patients achieved treatment success, far below the 90% target set by the WHO End TB Strategy (1, 2). While novel, shorter, all-oral regimens (e.g., BPaLM) have improved prospects for many patients (3), significant outcome disparities persist across populations.

This challenge is set against a backdrop of profound global demographic aging. By the late 2070s, the number of persons aged 65 years and older globally is projected to reach 2.2 billion, surpassing the number of children (under age 18) (4). Older adults face a heightened risk of developing TB due to immunosenescence, latent infection reactivation, and a higher prevalence of comorbidities such as diabetes and chronic kidney disease (5). Moreover, age-related physiological changes, including reduced hepatic and renal function, can impair drug metabolism and increase the risk of adverse reactions, complicating the management of toxic MDR-TB regimens (6). Consequently, older TB patients, particularly those with drug-resistant disease, experience higher rates of treatment failure, loss to follow-up, and mortality (7–9), further straining public health systems.

In China, this demographic shift is especially pronounced. The older population exceeded 190 million in 2020 (13.5% of the total) and is expected to surpass 30% by 2050 (10). China also bears a significant portion of the global MDR/RR-TB burden, accounting for an estimated 7.1% of incident cases, which translates to roughly 30,000 new MDR/RR-TB cases annually (1, 11). Despite remarkable progress in national TB control, evidenced by an annual incidence decline of 3.4% from 2010 to 2020 (12), older patients have not benefited equally. Reported treatment success rates for older RR-TB patients in China were only 37.2–65.7% (13, 14), which are considerably lower than those reported in other international studies on drug-resistant tuberculosis (>75%) (15, 16).

The care cascade framework is a powerful tool for quantifying gaps in health service delivery and identifying critical points of patient attrition. Previous cascade analyses in TB care have consistently identified the step between diagnosis and treatment initiation as a major point of failure (17), with 15–20% of diagnosed MDR/RR-TB patients never starting therapy (18, 19). A recent global review confirmed substantial pre-treatment losses but aggregated data across all age groups (20), while a provincial Chinese study highlighted significant attrition in an MDR/RR-TB cohort that also did not focus on older patients (21). Few studies have focused on the care cascade of older populations, and none have been conducted in the Chinese context with age-specific stratification for MDR/RR-TB. This lack of age-stratified analysis represents a critical knowledge gap. It obscures our understanding of how the unique biological, clinical, and social factors associated with aging—such as functional decline, social isolation, and economic insecurity—interact to influence outcomes along the MDR/RR-TB care pathway.

To address these gaps, we conducted a retrospective cohort study of older patients (≥65 years) with laboratory-confirmed MDR/RR-TB in Guangdong Province, a high-burden region in southern China. Our objectives were to quantify attrition at each stage of the care cascade, to identify key socioeconomic and health system determinants of treatment initiation and final outcomes, and to elucidate the specific and modifiable barriers contributing to the disproportionately poor outcomes in this vulnerable population. By providing age-specific evidence on where and why older patients are lost from care, this study aims to inform the development of geriatric-tailored interventions to improve treatment initiation, retention and success, thereby closing the gaps in the care cascade and ultimately advancing equity in global TB control.

## 2 Methods

### 2.1 Study design and data source

We conducted a retrospective cohort study that included all patients aged ≥65 years with laboratory-confirmed MDR/RR-TB in Guangdong Province, southern China, between January 2010 and December 2022. Guangdong is a densely populated, economically developed region with a substantial migrant population, providing a representative setting for examining the MDR/RR-TB care continuum. Patient-level data were extracted from the national Tuberculosis Information Management System (TIMS), a centralized, web-based national surveillance platform that serves as the cornerstone of China’s TB control program. For each eligible patient, anonymized records were obtained encompassing demographic characteristics (age, sex, occupation, migrant status, registration region), clinical presentation and diagnostic information (previous history of TB treatment, test method for confirming drug resistance, drug-resistance pattern), and treatment-related data (initiation status, follow-up assessments, final treatment duration and outcome).

### 2.2 Definitions

Local residency was defined by official household registration within the county; otherwise, patients were classified as domestic migrants. Registration regions were categorized as Pearl River Delta (PRD) or non-PRD within Guangdong Province, which comprises of 21 prefecture-level cities in southern China. The PRD region, a more developed area, is located in the central part of Guangdong and consists of nine cities (Guangzhou, Shenzhen, Foshan, Dongguan, Huizhou, Zhongshan, Zhuhai, Jiangmen, and Zhaoqing). The non-PRD region comprises the remaining twelve cities (Shantou, Shanwei, Jieyang, Chaozhou, Zhanjiang, Maoming, Yangjiang, Yunfu, Meizhou, Heyuan, Qingyuan, and Shaoguan). Previous history of TB treatment was defined as any prior anti-TB treatment lasting >30 days. The test method for confirming drug resistance was categorized as conventional drug-susceptibility testing (DST) or molecular DST (e.g., GeneXpert MTB/RIF). Drug-resistance pattern was defined as either RR-TB (rifampicin-resistant alone) or MDR-TB (resistant to at least isoniazid and rifampicin) (22). Treatment outcomes were defined according to the Technical Guidelines for Tuberculosis Prevention and Control in China (23). Cure was defined as completion of the prescribed treatment course without evidence of failure, plus at least three consecutive negative sputum cultures (≥30-day intervals) after the intensive phase. Treatment completion was defined as finishing the prescribed course without evidence of failure, but without achieving the above culture conversion. Cure or completion was classified as a successful outcome, consistent with WHO definitions. All other outcomes, including failure, death, and loss to follow-up, were grouped as poor treatment outcomes.

### 2.3 Care cascade construction

We constructed a systematic care cascade to visualize the treatment and management process of patients, which comprises four sequential stages and three inter-stage gaps. Stages represent the absolute number of individuals reaching each point along the care pathway:

Stage 1: Diagnosis of MDR/RR-TB (laboratory-confirmed).
Stage 2: Initiation of a WHO-recommended treatment regimen.
Stage 3: Remaining on treatment at the 6-month follow-up.
Stage 4: Success of treatment (including cure and completion of treatment).

Gaps represent attrition between successive stages:

Gap 1: Diagnosed but did not initiate treatment.
Gap 2: Initiated treatment but discontinued before 6 months.
Gap 3: Remained on treatment at 6 months but experienced poor treatment outcomes.

A Sankey diagram was generated using the web-based tool SankeyMATIC to visualize patient flow across the cascade (Figure 1).

**Figure 1:**
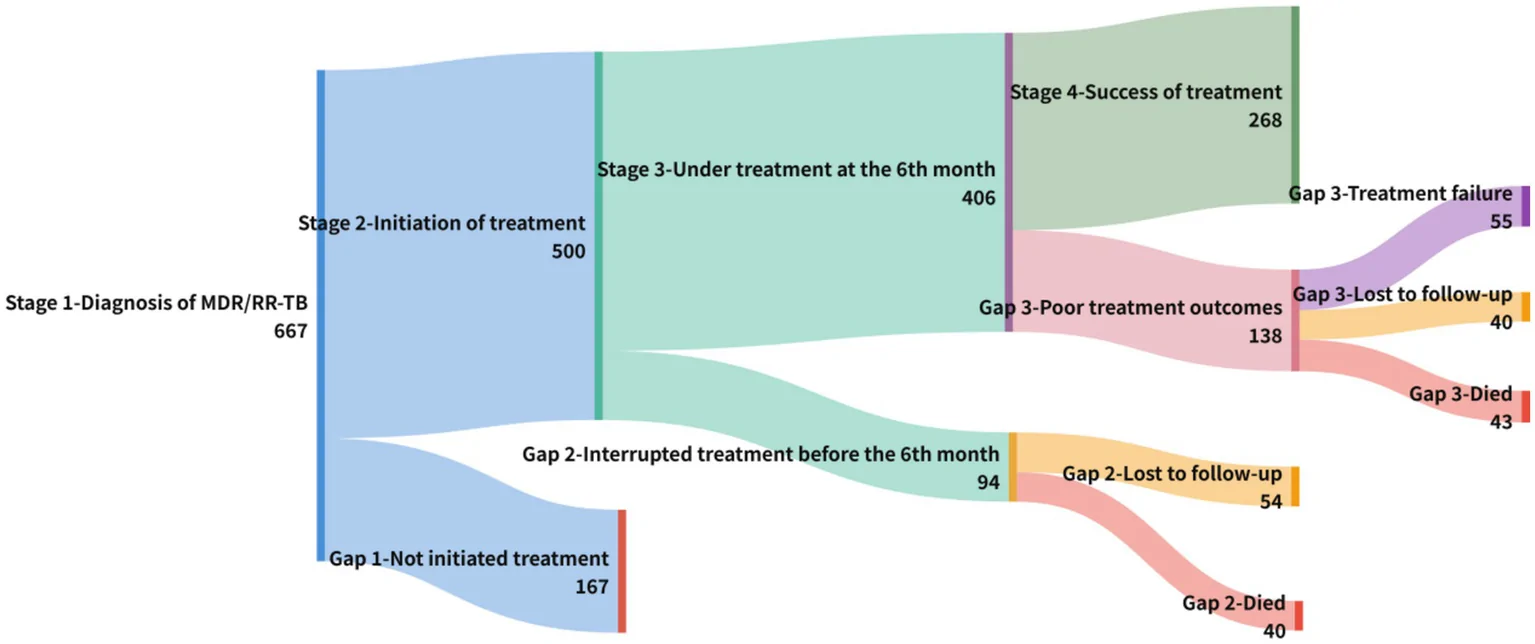
Treatment cascade for patients diagnosed with MDR/RR-TB.

### 2.4 Statistical analysis

Descriptive statistics were used to characterize the cohort and quantify the number and proportion of patients retained at each cascade stage. Categorical variables were presented as frequencies and percentages. Chi-square tests were applied to compare the characteristics of patients who did vs. those who did not complete successive cascade stages (e.g., initiated vs. did not initiate treatment). Although baseline attrition occurred during the treatment cascade, we retained these patients in the analytic model using imputation to handle missing endpoint data. For the covariates retained in the final analyses, simple imputation methods (e.g., mean imputation) were used for variables with <10% missing data. For variables with missing data between 10 and 50%, multiple imputation, regression estimation, and other methods were employed. Variables with missing data >50% were deleted. To identify independent factors associated with attrition at each major gap, we fitted separate multivariable logistic-regression models for two dependent variables: (1) not initiating MDR/RR-TB treatment, and (2) poor treatment outcomes among initiators. Covariates included in both models were age, sex, occupation, migrant status, previous history of TB treatment, test method and drug-resistance pattern. Results are presented as adjusted odds ratios (*a*ORs) with 95% confidence intervals (CIs). A two-tailed *p*-value of < 0.05 was considered statistically significant. All analyses were performed using R software (version 4.4.1; R Foundation for Statistical Computing).

## 3 Results

### 3.1 Patient characteristics and care cascade

A total of 667 older patients with laboratory-confirmed MDR/RR-TB were included in this retrospective cohort study (Table 1). The median age was 70 years (IQR 67–75), and the cohort was predominantly male (533, 79.91%). Most patients were non-migrants (529, 79.31%) and nearly one-third were workers/farmers (209, 31.33%). Over half were registered in the PRD region (400, 59.97%). The vast majority had a previous history of TB treatment (525, 78.71%). Drug resistance was primarily confirmed by conventional DST (489, 73.31%), with molecular DST was used in the remaining 178 (26.69%). MDR-TB accounted for 75.26% of all cases (502/667).

**Table 1:** Characteristics of MDR/RR-TB patients (*N* = 667).

Characteristic	Diagnosis of MDR/RR-TB (*n*, %)
Demographic and socioeconomic characteristics	
Age (years)	70 (67–75)
Sex	
Male	533 (79.91)
Female	134 (20.09)
Occupation	
Workers/Farmers	209 (31.33)
Others	458 (68.67)
Migrant	
No	529 (79.31)
Yes	138 (20.69)
Registration regions	
PRD	400 (59.97)
Non-PRD	267 (40.03)
Clinical presentation and diagnostic information	
Previous history of TB treatment	
No	142 (21.29)
Yes	525 (78.71)
Test method	
Conventional DST	489 (73.31)
Molecular biology DST	178 (26.69)
Drug resistance pattern	
RR-TB	165 (24.74)
MDR-TB	502 (75.26)

Figure 2 illustrates the care cascade with attrition rates at each stage. Of the 667 diagnosed patients, 74.96% (500/667) initiated MDR/RR-TB treatment. Among those who initiated, 406 (60.87% of diagnosed; 81.20% of treated) remained on treatment at the 6-month follow-up. Ultimately, only 268 patients (40.18% of diagnosed, 53.60% of treated) achieved cure or treatment completion. Among the 232 patients with poor outcomes, 98 (42.2%) died, 87 (37.5%) were lost to follow-up, and 47 (20.3%) experienced treatment failure.

**Figure 2:**
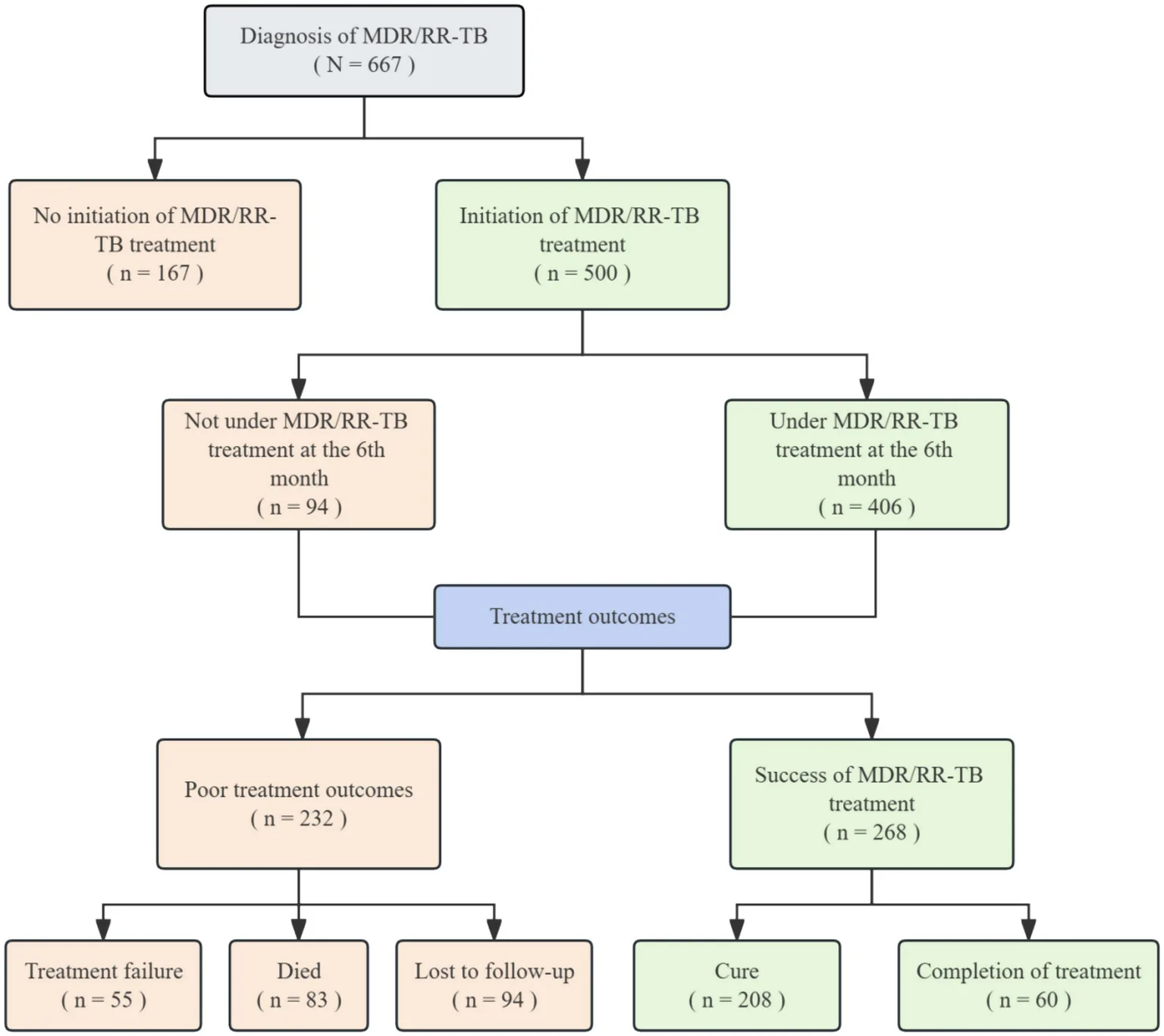
Flow chart of the treatment cascade for MDR/RR-TB patients.

### 3.2 Temporal trends of treatment initiation and treatment outcomes

Figure 3 and Supplementary Table S1 present the annual distribution of diagnosed older MDR/RR-TB cases, along with treatment initiation, 6-month retention, and treatment success rates from 2010 to 2022. As the annual number of diagnosed cases increased, treatment initiation rates rose substantially, from 55.6% (5/9) in 2010 to 96.0% (72/75) in 2022. 6-month retention rates remained relatively stable over time, with no clear temporal pattern. Treatment success rates fluctuated considerably during the early years (ranging from 28.6 to 60.0% between 2010 and 2015) but showed a more consistent upward trend from 2016 to 2022.

**Figure 3:**
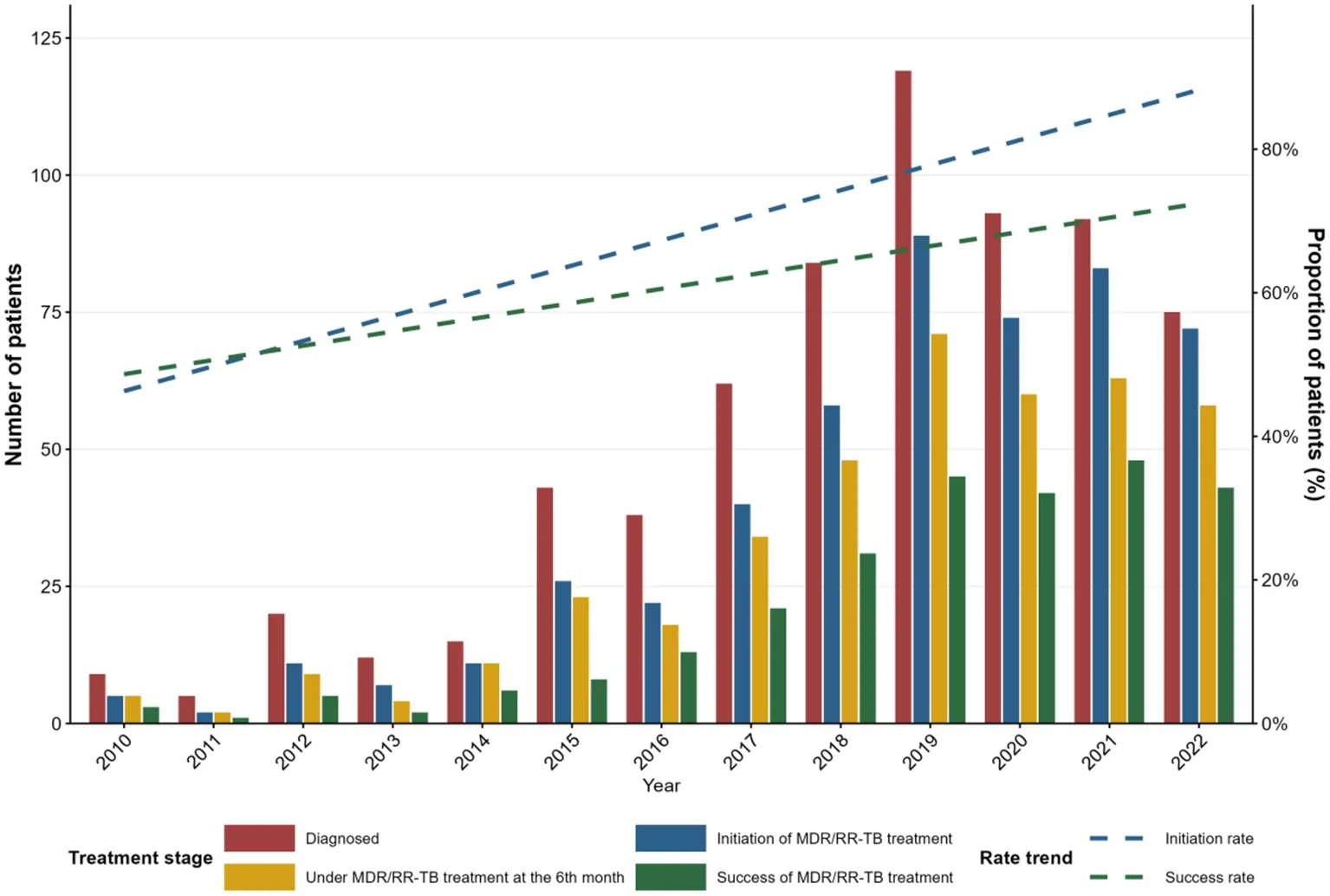
Temporal trends of treatment initiation and treatment outcomes among older patients with MDR/RR-TB.

Supplementary Table S2 and Supplementary Figure S1 present the results of the joinpoint regression analysis of treatment success rates. Among all potential breakpoints tested, the most significant change point was identified in 2016 (jump effect: 24.30, standard error: 9.73, *p* = 0.037), with a second notable change point in 2014 (jump effect: 25.15, standard error: 10.81, *p* = 0.048). The R^2^ values for these models were 0.559 and 0.607, respectively. The mean treatment success rate increased from 43.87% in the period preceding 2016 to 55.70% thereafter. Other candidate years (2012, 2017, 2015, 2020, 2018, 2021, 2019, and 2013) did not achieve statistical significance (all *p* > 0.05). These findings indicate that the most substantial improvement in treatment success occurred around 2016.

### 3.3 Comparison of patient characteristics through the care cascade

#### 3.3.1 Initiation of MDR/RR-TB treatment and retention of treatment at 6 months

Several characteristics demonstrated significant associations with failure to initiate treatment (Table 2). Notably, only 65.07% (136/209) of workers/farmers started treatment, compared with 79.48% (364/458) of patients with other occupations (*p* < 0.01). Patients who initiated treatment were younger than those who did not (median 70 vs. 71 years, *p* < 0.01). Initiation was also substantially higher among patients diagnosed by molecular DST (94.94%, 169/178) than by conventional DST (67.69%, 331/489; *p* < 0.01) and higher in RR-TB patients (85.45%, 141/165) than in MDR-TB patients (71.51%, 359/502; *p* = 0.01). Sex, migrant status, registration region, and previous TB treatment history showed no significant association with treatment initiation (all *p* > 0.05). Of the 500 patients who started treatment, 406 (81.2%) were retained at 6 months. Younger age was associated with higher retention (*p* < 0.01). No other demographic or clinical characteristics showed statistically significant association with 6-month retention (all *p* > 0.05).

**Table 2:** Comparison of the characteristics of patients in each cascade stage.

Characteristics	Patients who have initiated MDR/RR-TB treatment *n* (%)	Patients who have not initiated MDR/RR-TB treatment *n* (%)	*χ*^2^/W	*p* value	Under MDR/RR-TB treatment at the 6th month *n* (%)	Not under MDR/RR-TB treatment at the 6th month *n* (%)	*χ*^2^/W	*p* value	Success of MDR/RR-TB treatment *n* (%)	Poor treatment outcomes *n* (%)	*χ*^2^/W	*p* value
Age (years, IQR)	70 (66–74)	71 (68–77)	47785.5	<0.01	70 (66–74)	72 (68–77)	22636.0	<0.01	70 (66–73)	70 (67–76)	34584.5	0.03
Sex			0.43	0.51			0.25	0.62			2.18	0.14
Male	403 (75.61)	130 (24.39)			325 (80.65)	78 (19.35)			209 (51.86)	194 (48.14)		
Female	97 (72.39)	37 (27.61)			81 (83.51)	16 (16.49)			59 (60.82)	38 (39.18)		
Occupation			15.11	<0.01			0.28	0.60			2.22	0.14
Workers/Farmers	136 (65.07)	73 (34.93)			113 (83.09)	23 (16.91)			65 (47.79)	71 (52.21)		
Others	364 (79.48)	94 (20.52)			293 (80.49)	71 (19.51)			203 (55.77)	161 (44.23)		
Migrant			2.42	0.12			0.86	0.35			0.05	0.83
No	389 (73.53)	140 (26.47)			312 (80.21)	77 (19.79)			210 (53.98)	179 (46.02)		
Yes	111 (80.43)	27 (19.57)			94 (84.68)	17 (15.32)			58 (52.25)	53 (47.75)		
Registration region			0.23	0.63			0.35	0.55			0.56	0.45
PRD	303 (75.75)	97 (24.25)			243 (80.2)	60 (19.8)			167 (55.12)	136 (44.88)		
Non-PRD	197 (73.78)	70 (26.22)			163 (82.74)	34 (17.26)			101 (51.27)	96 (48.73)		
Previous history of TB treatment			0.04	0.84			0.12	0.73			3.28	0.07
No	105 (73.94)	37 (26.06)			87 (82.86)	18 (17.14)			65 (61.9)	40 (38.1)		
Yes	395 (75.24)	130 (24.76)			319 (80.76)	76 (19.24)			203 (51.39)	192 (48.61)		
Test method			50.21	<0.01			0.81	0.37			6.00	<0.01
Conventional DST	331 (67.69)	158 (32.31)			273 (82.48)	58 (17.52)			164 (49.55)	167 (50.45)		
Molecular Biology DST	169 (94.94)	9 (5.06)			133 (78.7)	36 (21.3)			104 (61.54)	65 (38.46)		
Drug resistance pattern			12.13	0.01			0.58	0.45			2.49	0.11
RR-TB	141 (85.45)	24 (14.55)			111 (78.72)	30 (21.28)			84 (59.57)	57 (40.43)		
MDR-TB	359 (71.51)	143 (28.49)			295 (82.17)	64 (17.83)			184 (51.25)	175 (48.75)		
Diagnosis year												
Pre-2016	62 (59.62)	42 (40.38)	15.46	<0.01	54 (87.10)	8 (12.90)	1.61	0.20	25 (40.32)	37 (59.68)	5.02	0.03
Post-2016	438 (77.80)	125 (22.20)			352 (80.37)	86 (19.63)			243 (55.48)	195 (44.52)		

#### 3.3.2 Treatment outcomes

Among the 500 patients who initiated treatment, 268 (53.6%) achieved cure or treatment completion. Patients with successful treatment outcomes were younger than those with poor outcomes (*p* = 0.03). The test method was the only factor significantly associated with the final treatment outcome. Success rates were 61.54% (104/169) for patients diagnosed by molecular DST vs. 49.55% (164/331) for those diagnosed by conventional DST (*p* < 0.01). No other demographic or clinical factors showed significant association with treatment outcomes in the bivariate analysis (all *p* > 0.05).

### 3.4 Multivariable analysis of cascade gaps

#### 3.4.1 Failure to initiate treatment and retention of treatment at 6 months

In multivariable analysis (Figure 4), workers/farmers (*a*OR = 1.87, 95% CI: 1.24–2.84), diagnosis of MDR/RR-TB by conventional DST (*a*OR = 9.34, 95% CI: 4.52–21.64) and diagnosed before 2016 (*a*OR = 1.91, 95% CI: 1.18–3.08) were independent risk factors for not initiating treatment. Previous history of TB treatment (*a*OR = 0.42, 95% CI:0.25–0.72) was protective. Each additional year of age also increased the odds of non-initiation (*a*OR per year = 1.05, 95% CI: 1.01–1.08) and non-retention of treatment at 6 months (*a*OR per year = 1.06, 95% CI: 1.02–1.10).

**Figure 4:**
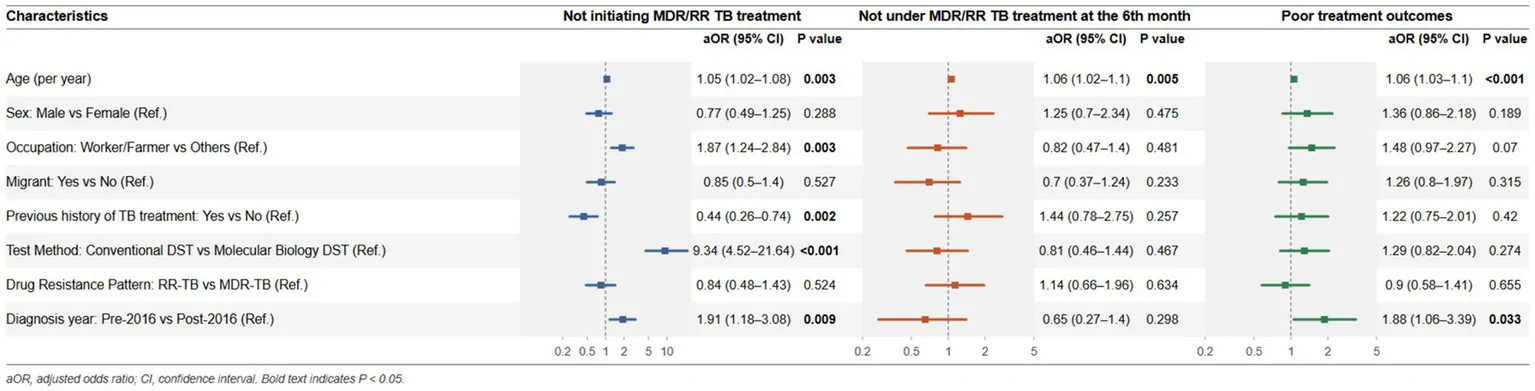
Factors associated with failure to initiate treatment, failure to retain treatment at 6 months, and poor treatment outcomes.

#### 3.4.2 Poor treatment outcomes

Older age (*a*OR = 1.06 per year increase, 95% CI: 1.02–1.09) and diagnosed before 2016 (*a*OR = 1.88, 95% CI: 1.06–3.39) were risk factors for poor outcomes. No other variables showed statistically significant associations with poor treatment outcomes (all *p* > 0.05; Figure 4).

## 4 Discussion

This retrospective cohort study provides one of the first detailed analyses of the treatment cascade specifically for older patients (≥65 years) with MDR/RR-TB in Guangdong Province, China, a high-burden region for TB. Our findings reveal substantial attrition across the care pathway, with only 40.18% of the diagnosed cohort achieving treatment success. Cascade analysis highlighted three critical gaps: 25.04% of diagnosed patients failed to initiate treatment (Gap 1), 18.80% of those who initiated treatment dropped out by 6 months (Gap 2), and 46.40% of those who initiated treatment experienced poor outcomes (Gap 3). Although this pattern of pre- and post-initiation losses aligns with cascade studies in other settings (18, 19, 24, 25), our age-stratified data uniquely quantify the heightened vulnerability of the older adults within the MDR/RR-TB care system and identify key demographic, clinical, and system-level factors associated with these disparities.

Our study spans a 13-year period (2010–2022) during which MDR/RR-TB diagnosis and treatment in China underwent substantial transformation. The progressive introduction and scale-up of molecular diagnostics (e.g., GeneXpert MTB/RIF), the transition from injectable-containing longer regimens to all-oral shorter regimens, and the strengthening of programmatic management capacity all occurred within this window (26, 27). The temporal trends analysis identified 2016 as the most significant breakpoint, with mean treatment success increasing from 43.87% before 2016 to 55.70% thereafter. Therefore, we divided the study period into pre-2016 and post-2016 eras and incorporated this time stratification into all multivariable models to account for potential confounding by temporal changes.

The most pronounced loss occurred at the diagnosis-to-treatment transition, with 25.04% of older patients never initiating MDR/RR-TB therapy. This rate is comparable to the 25–28% pre-treatment attrition reported in MDR/RR-TB cohorts from China and other settings that encompass all age groups (19, 21). Multivariable analysis identified workers/farmers, reliance on conventional DST, diagnosis before 2016, and older age as risk factors for failure to initiate treatment, while prior TB treatment history was protective. The low initiation rate among workers/farmers (65.07%) illustrates how occupational vulnerability, often overlooked in all-age studies (21), disproportionately exacerbates attrition in older adults, likely due to structural barriers such as limited healthcare access and financial constraints (28). The strong association between conventional DST and non-initiation may reflect a combination of factors, including diagnostic delays, limited laboratory capacity, and other system-level constraints that are more prevalent in settings where conventional DST is still used. Previous studies have suggested that diagnostic delays contribute to treatment refusal or delay (29, 30), while rapid molecular assays enable faster and more accurate diagnosis, potentially allowing for earlier initiation of tailored regimens (31). Conversely, the protective effect of prior TB treatment experience likely stems from greater familiarity with TB care pathways, which reduces barriers to re-engagement (17).

In this study, the overall treatment success rate was 53.60% among those who initiated treatment. Increasing age remained the sole independent risk factor for poor outcomes (*a*OR = 1.06 per year, 95% CI: 1.02–1.09), consistent with physiological changes (e.g., impaired drug metabolism, immunosenescence) and higher comorbidity burdens in advanced age (32, 33). However, this finding requires careful temporal contextualization when compared with global estimates. According to the WHO Global Tuberculosis Report 2025, the global MDR/RR-TB treatment success rate has improved from 63% in 2021 to 71% in 2024 (1), an improvement attributed to the increasing adoption of novel all-oral short-course regimens such as BPaLM (bedaquiline–pretomanid–linezolid–moxifloxacin). These contemporary global estimates predominantly reflect treatment outcomes achieved with newer regimens in cohorts treated after 2020. In contrast, our cohort spans 2010–2022, during which the majority of patients received older, injectable-containing longer regimens that were the standard of care for most of the study period. When stratified by calendar period, treatment success rates in our cohort increased from 43.87% before 2016 to 55.70% after 2016, with the post-2016 rate approaching 66%. Nevertheless, even the most recent period in our cohort remains substantially below the WHO’s End TB target of 90% (34), underscoring the persistent challenges faced by older patients that extend beyond regimen efficacy alone.

Leveraging a large population-based surveillance system with age-stratified cascade analysis, our findings from this high-burden setting offer evidence to inform targeted interventions. Firstly, accelerate the replacement of conventional DST with molecular diagnostics for older presumptive MDR/RR-TB patients and expedite result reporting (within 48 h) to reduce diagnostic delays and improve treatment engagement. This recommendation aligns with WHO guidelines for high-risk groups (35). Secondly, provide tailored support for workers/farmers, such as financial subsidies, home-based care, and flexible treatment schedules, to overcome the structural barriers identified as major drivers of non-initiation in this subgroup. Thirdly, strengthen age-adapted follow-up. Routine care should incorporate comorbidity screening, adverse reaction monitoring, and low-threshold geriatrician referral. Generalizability to other settings requires caution given regional heterogeneity.

Several limitations should be acknowledged. First, as a retrospective analysis, we were unable to capture unmeasured confounders such as comorbidities (e.g., diabetes, chronic kidney disease), functional status, or caregiver support, which may bias estimates. Second, the lack of data on diagnostic delay limited our ability to attribute non-initiation to conventional DST. Prospective studies with time-to-diagnosis measures are needed. Third, separate multivariable analyses for each poor outcome component were not conducted. Future larger studies should identify component-specific risk factors, which would inform more targeted interventions for this vulnerable population.

## 5 Conclusion

Older patients with MDR/RR-TB experience substantial attrition throughout the care cascade, driven predominantly by diagnostic delays, occupational disadvantages, and age-related physiological vulnerabilities. This study provides the first age-stratified cascade analysis for this population in China and demonstrates that targeted interventions, including scaling up rapid molecular diagnostics, implementing tailored support for high-risk occupational groups, strengthening age-adapted follow-up, and expanding active case finding, are essential to close critical gaps in geriatric MDR/RR-TB care. As the global population ages, these findings highlight the urgent need to integrate age-specific considerations into national and global TB control strategies to advance toward the WHO’s End TB targets.

## Data Availability

The original contributions presented in the study are included in the article/Supplementary material, further inquiries can be directed to the corresponding authors.
