# Supplementary material for: Severe pre-treatment attrition in the care cascade: clinical outcomes and risk factors among older MDR/RR-TB patients in China
Source: Front Public Health. 2026 Sep 16;14:1883068. doi: 10.3389/fpubh.2026.1883068 (PMC13624063; doi:10.3389/fpubh.2026.1883068)
Supplement: Supplementary file 1 [file Supplementary_file_1.docx]

**Supplementary materials**

**Table S1. Temporal trends of treatment initiation and treatment outcomes among elderly patients with MDR/RR-TB**

| Years | Diagnosis of MDR/RR-TB (n) | Initiation of MDR/RR-TB treatment (n, %) | Under MDR/RR-TB treatment at the 6^th^ month (n, %) | Success of MDR/RR-TB treatment (n, %) |
| --- | --- | --- | --- | --- |
| 2010 | 9 | 5 (55.6) | 5 (100) | 3 (60) |
| 2011 | 5 | 2 (40) | 2 (100) | 1 (50) |
| 2012 | 20 | 11 (55) | 9 (81.8) | 5 (45.5) |
| 2013 | 12 | 7 (58.3) | 4 (57.1) | 2 (28.6) |
| 2014 | 15 | 11 (73.3) | 11 (100) | 6 (54.5) |
| 2015 | 43 | 26 (60.5) | 23 (88.5) | 8 (30.8) |
| 2016 | 38 | 22 (57.9) | 18 (81.8) | 13 (59.1) |
| 2017 | 62 | 40 (64.5) | 34 (85) | 21 (52.5) |
| 2018 | 84 | 58 (69) | 48 (82.8) | 31 (53.4) |
| 2019 | 119 | 89 (74.8) | 71 (79.8) | 45 (50.6) |
| 2020 | 93 | 74 (79.6) | 60 (81.1) | 42 (56.8) |
| 2021 | 92 | 83 (90.2) | 63 (75.9) | 48 (57.8) |
| 2022 | 75 | 72 (96) | 58 (80.6) | 43 (59.7) |

**Table S2. Joinpoint regression analysis of treatment success rates**

| **Cutoff_year** | **Jump_effect** | **P_value** | **R_squared** |
| --- | --- | --- | --- |
| 2016 | 24.30 | 0.037 | 0.559 |
| 2014 | 25.15 | 0.048 | 0.607 |
| 2012 | -24.64 | 0.050 | 0.450 |
| 2017 | 8.68 | 0.503 | 0.236 |
| 2015 | 6.11 | 0.641 | 0.341 |
| 2020 | 6.29 | 0.644 | 0.182 |
| 2018 | 5.11 | 0.695 | 0.194 |
| 2021 | 4.68 | 0.744 | 0.160 |
| 2019 | 2.35 | 0.859 | 0.177 |
| 2013 | 1.05 | 0.946 | 0.491 |

**Figure S1. Joinpoint regression analysis of treatment success rates**.
